# Identification of a stool long non‐coding RNAs panel as a potential biomarker for early detection of colorectal cancer

**DOI:** 10.1002/jcla.23601

**Published:** 2020-10-23

**Authors:** Ehsan Gharib, Ehsan Nazemalhosseini‐Mojarad, Kaveh Baghdar, Zahra Nayeri, Hossein Sadeghi, Sama Rezasoltani, Arezo Jamshidi‐Fard, Pegah Larki, Amir Sadeghi, Mehrdad Hashemi, Hamid Asadzadeh Aghdaei

**Affiliations:** ^1^ Basic and Molecular Epidemiology of Gastrointestinal Disorders Research Center Research Institute for Gastroenterology and Liver Diseases Shahid Beheshti University of Medical Sciences Tehran Iran; ^2^ Gastroenterology and Liver Diseases Research Center Research Institute for Gastroenterology and Liver Diseases Shahid Beheshti University of Medical Sciences Tehran Iran; ^3^ Molecular Genetics Department Genomic Research Center Shahid Beheshti University of Medical Sciences Tehran Iran; ^4^ Department of Genetics Faculty of Advanced Science and Technology Tehran Medical Sciences Islamic Azad University Tehran Iran

**Keywords:** biomarker, colorectal cancer, fecal colonocytes, gene expression, long non‐coding RNAs

## Abstract

**Background:**

The feces of colorectal cancer (CRC) patients contain tumor colonocytes, which constantly shed into the lumen area. Therefore, stool evaluation can be considered as a rapid and low‐risk way to directly determine the colon and rectum status. As long non‐coding RNAs (lncRNAs) alterations are important in cancer cells fate regulation, we aimed to assess the level of a panel of cancer‐related lncRNAs in fecal colonocytes.

**Methods:**

The population study consisted of 150 subjects, including a training set, a validation set, and a group of 30 colon polyps. The expression levels of lncRNAs were evaluated by quantitative real‐time PCR (qRT‐PCR). The NPInetr and EnrichR tools were used to identify the interactions and functions of lncRNAs.

**Results:**

A total of 10 significantly dysregulated lncRNAs, including CCAT1, CCAT2, H19, HOTAIR, HULC, MALAT1, PCAT1, MEG3, PTENP1, and TUSC7, were chosen for designing a predictive panel. The diagnostic performance of the panel in distinguishing CRCs from the healthy group was AUC: 0.8554 in the training set and 0.8465 in the validation set. The AUC for early CRCs (I‐II TNM stages) was 0.8554 in the training set and 0.8465 in the validation set, and for advanced CRCs (III‐IV TNM stages) were 0.9281 in the training set and 0.9236 in the validation set. The corresponding AUC for CRCs vs polyps were 0.9228 (I‐IV TNM stages), 0.9042 (I‐II TNM stages), and 0.9362 (III‐IV TNM stages).

**Conclusions:**

These data represented the application of analysis of fecal colonocytes lncRNAs in early detection of CRC.

## Graphical abstract

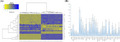

## 1 INTRODUCTION

CRC has become one of the first priorities of the World Health Organization (WHO) for mass screening due to high morbidity and mortality rates. It develops from a slow progressive premalignant lesion (the adenomatous polyp), which can readily be removed by an accurate diagnosis.^1^ Based on the risk level of the malignancy, screening approaches for CRC patients are divided into two main categories: average‐risk population and high‐risk population. Each of these categories is targeted by a different screening program.^2^ According to WHO guidelines, both categories should have been monitored constantly using standard screening methods such as colonoscopy.^1^, ^2^ However, given the various disadvantages of these technics, current investigations are being taken into consideration for substituting noninvasive, inexpensive screening methods with more specificity and sensitivity.^3^ There are ongoing optimizations to simplify the process of identifying new biomarkers from body specimens such as stool, plasma, and urine.^4^ Long non‐coding RNAs (lncRNAs) are an important class of ncRNAs that have a huge impact on the cancer progress. These RNAs are transcribed by RNA polymerase II, with a length of 200 nt or more, from different regions of the genome, including intronic and intergenic sites.^5^, ^6^ Considering this point, it has been inferred that lncRNA transcription usually does not depend on the presence of the open reading frames and it has been estimated that the human genome contains more than 15 000 lncRNAs‐related genes that could produce over 23 000 functional lncRNAs.^7^ This large proportion brings the idea that this class of ncRNAs may contribute to a wide variety of regulatory activities such as transcriptional activation/repression, epigenetic regulation, nuclear remodeling, mRNAs stability/degradation, and the microRNA (miRNA) sponge.^5^, ^7^ Through these mechanisms, lncRNAs are involved in multiple cancer‐related signaling cascades and provoke tumor development or suppression.^4^ Furthermore, lncRNAs might be used as biomarkers for the early detection of metastasis in CRC and are regarded as novel biomarkers and therapeutic targets for CRC patients.^8^

The diagnostic value of lncRNAs in CRC has not been completely examined due to sampling issues, especially at early stages of the disease. Considering this point that most of the cancer detections are happening in advanced stages, identification of cancer‐related biomarkers that actually initiated the malignancy is challenging. Routine tests on tissue samples for the early detection of colorectal cancer (CRC) have some problems such as invasiveness, lack of evaluation by an expert pathologist, cost‐intensive, and time‐consuming. So, we need to explore other biological samples such as blood, urine, and stool, which are easier to collect and analyze. Among these samples, the stool takes priority, passing throughout the colon and rectal regions and could carry cancer colonic cells (cancer colonocytes). Fecal collection is also easy, inexpensive, noninvasive, and accessible from all ages. Previous investigations proved the existence of the miRNAs in stool samples.^9^ However, to the best of our knowledge, no report has been published on the analysis of fecal lncRNAs expression levels. Considering the values of stool samples in the characterization of colon disorders, in this study, we aimed to track the alteration of the expression pattern of 30 known cancer‐related lncRNAs in human feces from healthy status to advanced carcinoma. The results of this investigation introduced the human fecal colonocytes as a proper source of lncRNAs for CRC analysis.

## 2 MATERIAL AND METHODS

### 2.1 Subjects

The population study consisted of 150 individuals including 60 CRC patients, 60 non‐cancer individuals, and a group of 30 individuals with colon polyps who were referred to the Taleghani Hospital, Tehran, Iran. They were divided into three cohorts: 1‐Training group (30 CRC and 30 normal), 2‐Validation group (30 CRC and 30 normal), and 3‐Examination and comparison of the final lncRNA panel; 30 CRC patients from the validation cohort and 30 individuals with colon polyps. All cases had been diagnosed and approved by the Gastroenterology and Liver Disease Research Institute (RCGLD), Shahid Beheshti University of Medical Sciences, Tehran, Iran, during the years 2010‐2017. The mean age of the population was 54 years. This study was conducted in accordance with the ethical principles of the World Medical Association's Declaration of Helsinki and approved by the Medical Ethical Committee of RCGLD, Tehran, Iran (Ethical code: IR.SBMU.RIGLD.REC.1397.949). The clinical features of the studied population are demonstrated in Table 1.

**Table 1:** Basic characteristics of the studied population

Variable	Training set	Validation set	*P* value
Healthy count (%)			
Sex			.8933
Male	17 (65.7)	15 (50)	
Female	13 (43.3)	15 (50)	
Age (y)			.7839
Mean + SD	42 ± 12	43 ± 11	
Polyp count (%)			
Sex			–
Male	–	14 (53.3)	
Female	–	16 (46.7)	
Age (y)			–
Mean + SD	–	42 ± 11	
Colorectal cancer count (%)			
Sex			
Male	16 (53.3)	13 (43.3)	.8241
Female	14 (46.7)	17 (65.7)	
Age (y)			.968
Mean + SD	65 ± 14	65 + 13	
TNM stage			.6055
I	5 (16.7)	6 (20)	
II	9 (30)	8 (26.7)	
III	7 (23.3)	10 (33.3)	
IV	9 (30)	6 (20)	
Healthy vs CRC (*P* value^2^)			
Sex	0.8295	0.8111	
Age	<0.001	<0.001	
Polyp vs CRC (*P* value^2^)			
Sex	–	0.9707	
Age	–	<0.001	

### 2.2 Sample processing and RNA extraction

An overall 20 g of fecal samples was taken from each candidate over a month. Using a swab, the samples were collected from either the stools’ mucinous region, as a rich source of colonocytes,^10^ or non‐mucinous areas, for evaluating the entire colon status. The collections were immediately dissolved in RNALater buffer (2 mL/g) and stored at −80°C for future analysis. One millilitre of patient dissolved stool was mixed with 3 mL of buffer containing 10 mmol/L Tris HCl (pH 7.4), 200 mmol/L NaCl, and 1 mmol/L EDTA and vortexed vigorously for 3 minutes. The mixture was centrifuged for 5 minutes at 12 000 *g*. The supernatants were transferred to the miRNeasy Mini Kit columns (QIAGEN) and preceded according to the manufacturer's protocol. To avoid genomic DNA contamination, RNA samples were treated with DNase I for 1 hour and examined by 1% agarose gel electrophoresis to evaluate RNA integrity. Additionally, the RNA concentration was estimated using the NanoDrop^®^ ND‐1000 spectrophotometer (Thermo Fisher Scientific). The RNA purity was evaluated according to the A260/A280 ratio.

### 2.3 Reverse transcription and PCR amplification

To ensure the absence of any possible contamination, samples were evaluated by the PCR method. A total amount of 1 μg DNase I‐treated RNA per sample was reverse‐transcribed with the QuantiTect Rev. Transcription Kit (QIAGEN) with random hexamer primers. The PCR reaction was performed using the Taq PCR Master Mix Kit (QIAGEN). The cDNA samples were amplified with an initial denaturation at 94°C for 3 minutes followed by 35 cycles each at 94°C for 60 seconds, 60°C for 45 seconds, and 72°C for 60 seconds with a final extension step at 72°C for 10 minutes. The PCR products were verified through 1% agarose gel electrophoresis.

### 2.4 Quantitative real‐time PCR (qRT‐PCR)

The qRT‐PCR was performed on the 7500 Real‐Time PCR System (Applied Biosystems) using the QuantiTect SYBR Green PCR Kit (QIAGEN). The relative abundance of targets expression was determined by normalizing to reference genes (18S rRNA, GAPDH, U6) using the 2^−∆∆CT^ method. The primer sequences are demonstrated in Table 2.

**Table 2:** The primer sequences of examined genes

Ensemble ID	Gene name	Chromosome (GRCh38)	Primers
ENSG00000240498	ANRIL	Chr9: 21994778‐22121097	(F) 5′‐CCGCTCCCCTATTCCCCTTA‐3′ (R) 5′‐CCTGATTGGCGGATAGAGCA‐3′
ENSG00000278768	BACE1‐AS	Chr11: 117290874‐117293571	(F) 5′‐GAAGGGTCTAAGTGCAGACATCTT‐3′ (R) 5′‐AGGGAGGCGGTGAGAGT‐3′
ENSG00000278910	BANCR	Chr9: 69296682‐69306977	(F) 5′‐ACAGGACTCCATGGCAAACG‐3′ (R) 5′‐ATGAAGAAAGCCTGGTGCAGT‐3′
ENSG00000270419	CAHM	Chr6: 163413065‐163413960	(F) 5′‐AGGGGAGCGTCAGTCGTGCT‐3′ (R) 5′‐TGCGGCTTCATTCCCTCACGG‐3′
ENSG00000177640	CASC2	Chr10: 118046279‐118210153	(F) 5′‐GCACATTGGACGGTGTTTCC‐3′ (R) 5′‐CCCAGTCCTTCACAGGTCAC‐3′
ENSG00000247844	CCAT1	Chr8: 127207866‐127219088	(F) 5′‐CATTGGGAAAGGTGCCGAGA‐3′ (R) 5′‐ACGCTTAGCCATACAGAGCC‐3′
ENSG00000280997	CCAT2	Chr8: 127400399‐127402150	(F) 5′‐CCCTGGTCAAATTGCTTAACCT‐3′ (R) 5′‐TTATTCGTCCCTCTGTTTTATGGAT‐3′
ENSG00000245694	CRNDE	Chr16: 54918863‐54929189	(F) 5′‐AAATCAAAGTGCTCGAGTGGT‐3′ (R) 5′‐ACCTTCTTCTGCGTGACAAC‐3′
ENSG00000226950	DANCR	Chr4: 52712404‐52720351	(F) 5′‐CTTGTAGCAACCACGTGTCC‐3′ (R) 5′‐GCAGCCTGTCCCTAACAGAAT‐3′
ENSG00000230590	FTX	ChrX: 73963955‐74293574	(F) 5′‐CAAAGCTGGTCCTGTGCCTG‐3′ (R) 5′‐ATTGAGTGTGGCATCACCTCC‐3′
ENSG00000266835	GAPLINC	Chr18: 3466250‐3478978	(F) 5′‐TCCCAGGCATCAGGTGTGAA‐3′ (R) 5′‐ACACATCACTGTAAACGTGCCT‐3′
ENSG00000234741	GAS5	Chr1: 173863900‐173868882	(F) 5′‐CTTGCCTGGACCAGCTTAAT‐3′ (R) 5′‐CAAGCCGACTCTCCATACCT‐3′
ENSG00000281189	GHET1	Chr7: 148987527‐148989432	(F) 5′‐TGTAAAGGTGCAGGCAAGGG‐3′ (R) 5′‐TGCTTTTCCATTGGCTTGGG‐3′
ENSG00000130600	H19	Chr11: 1995163‐2001470	(F) 5′‐GCAAGAAGCGGGTCTGTTT‐3′ (R) 5′‐GCTGGGTAGCACCATTTCTT‐3′
ENSG00000228630	HOTAIR	Chr12: 53962308‐53974956	(F) 5′‐GGCGGATGCAAGTTAATAAAAC‐3′ (R) 5′‐TACGCCTGAGTGTTCACGAG‐3′
ENSG00000243766	HOTTIP	Chr7: 27198575‐27207259	(F) 5′‐CCTAAAGCCACGCTTCTTTG‐3′ (R) 5′‐TGCAGGCTGGAGATCCTACT‐3′
ENSG00000251164	HULC	Chr6: 8653558‐8653797	(F) 5′‐ATCTGCAAGCCAGGAAGAGTC‐3′ (R) 5′‐CTTGCTTGATGCTTTGGTCTGT‐3′
ENSG00000269821	KCNQ1OT1	Chr11: 2608328‐2699994	(F) 5′‐CTTTGCAGCAACCTCCTTGT‐3′ (R) 5′‐TGGGGTGAGGGATCTGAA‐3′
ENSG00000231721	LINC‐PINT	Chr7: 130941760‐131110176	(F) 5′‐GAACGAGGCAAGGAGCTAAA‐3′ (R) 5′‐AGCAAGGCAGAGAAACTCCA‐3′
ENSG00000258609	LINC‐ROR	Chr18: 57054559‐57072119	(F) 5′‐TATAGTTCTTCCAGGTCTCAGG‐3′ (R) 5′‐CTTTCGAGGTTATCAGGGTG‐3′
ENSG00000281183	lncRNA‐LET	Chr15: 73567012‐73569294	(F) 5′‐CCTTCCTGACAGCCAGTGTG‐3′ (R) 5′‐CAGAATGGAAATACTGGAGCAAG‐3′
ENSG00000251562	MALAT1	Chr11: 65497762‐65506516	(F) 5′‐AACGCAGACGAAAATGGAAAGA‐3′ (R) 5′‐CCTTCTAACTTCTGCACCACCAGA‐3′
ENSG00000214548	MEG3	Chr14: 100779410‐100861031	(F) 5′‐CTGCCCATCTACACCTCACG‐3′ (R) 5′‐TGTTGGTGGGATCCAGGAAA‐3′
ENSG00000245532	NEAT1	Chr11: 65422774‐65445540	(F) 5′‐CTTCCTCCCTTTAACTTATCCATTC‐3′ (R) 5′‐CTCTTCCTCCACCATTACCAACAATAC‐3′
ENSG00000253438	PCAT1	Chr8: 126847055‐127021014	(F) 5′‐TTGTGGAAGCCCCGCAAGGCCTGAA‐3′ (R) 5′‐TGTGGGGCCTGCACTGGCACTT‐3′
ENSG00000237984	PTENP1	Chr9: 33673504‐33677499	(F) 5′‐TCAGAACATGGCATACACCAA‐3′ (R) 5′‐TGATGACGTCCGATTTTTCA‐3′
ENSG00000249859	PVT1	Chr8: 127794533‐128101253	(F) 5′‐TTGCTTCTCCTGTTGCTGCT‐3′ (R) 5′‐GCTGGGTCTTCATCCTGAGT‐3′
ENSG00000253352	TUG1	Chr22: 30970677‐30979395	(F) 5′‐TAGCAGTTCCCCAATCCTTG‐3′ (R) 5′‐CACAAATTCCCATCATTCCC‐3′
ENSG00000243197	TUSC7	Chr3: 116709235‐116723581	(F) 5′‐CACTGCCTATGTGCACGACT‐3′ (R) 5′‐AGAGTCCGGCAAGAAGAACA‐3′
ENSG00000214049	UCA1	Chr19: 15828961‐15836320	(F) 5′‐CTCTCCATTGGGTTCACCATTC‐3′ (R) 5′‐GCGGCAGGTCTTAAGAGATGAG‐3′
ENSG00000111640	GAPDH	Chr12: 6533927‐6538374	(F) 5′‐GCTCTCTGCTCCTCCTGTTC‐3′ (R) 5′‐ACGACCAAATCCGTTGACTC‐3′
	U6	Chr15: 68132278‐ 68132383	(F) 5′‐CTCGCTTCGGCAGCACA‐3′ (R) 5′‐AACGCTTCACGAATTTGCGT‐3′
	18S rRNA	Unplaced	(F) 5′‐GAGAAACGGCTACCACATCC‐3′ (R) 5′‐TTTTTCGTCACTACCTCCCC‐3′

### 2.5 Function enrichment analysis

The functional interactions between candidate lncRNAs and biomolecules (proteins, RNAs, and DNAs) were identified by NPInter (http://www.bioinfo.org/NPInter, Version 3.0). The statistical enrichment of lncRNAs targets was analyzed using the Kyoto Encyclopedia of Genes and Genomes (KEGG) pathways annotation through the Enrichr database (http://amp.pharm.mssm.edu/Enrichr, 2018). The KEGG pathways were considered significantly enriched if *P*‐value < .05. Networks were demonstrated using Cytoscape version 3.6.1.^11^ Those genes with functional relationship were depicted as cluster networks by the Cytoscape plugin ClueGO.^12^ Furthermore, by using the Cytoscape plugin Cyto‐Hubba, we identified the subjected network hubs.^13^

### 2.6 Statistical analysis

To assess the differences in the lncRNAs expression level, we used the Mann‐Whitney *U* test. The diagnostic lncRNA markers were selected in the training datasets using logistic regression. The receiver operation characteristic (ROC) curve was established to estimate the diagnostic values of the lncRNA panel. All data are represented as mean ± SD, and *P* < .05 was considered statistically significant. All statistical analyses were performed using SPSS Statistics Software version 22 (IBM).

## 3 RESULTS

### 3.1 Quality assessment of isolated RNA from the stool

In order to confirm the non‐contamination of the sample with other organisms’ RNAs, the expression level of 18S RNA as the internal control was measured along with bacterial 16S RNA and chloroplast RuBisCO by PCR, and the amplifications were examined by gel electrophoresis.

### 3.2 Identification of differentially expressed lncRNAs (DElncRNAs) in training set

**Figure 1:**
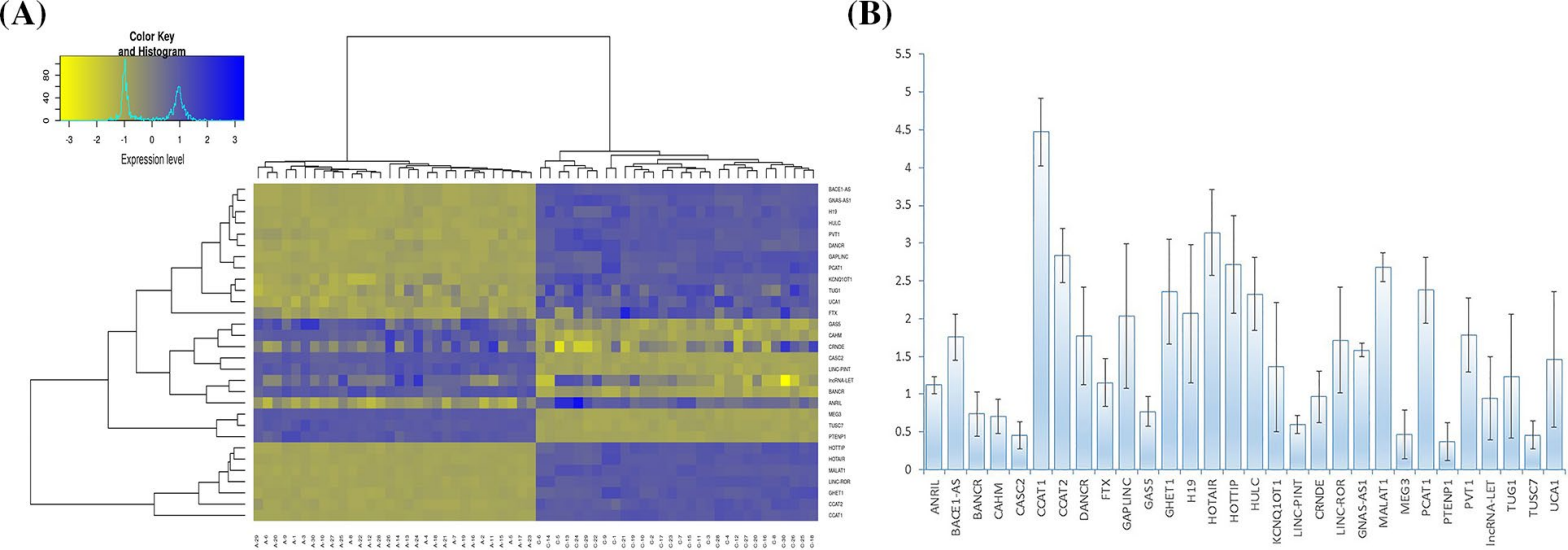
The expression profile of fecal lncRNAs in the training set. A, Hierarchical clustering of fecal lncRNAs expression pattern of training set. The hierarchical clustering was designed with 30 differently expressed lncRNAs in 30 colorectal cancer (CRC) cases and 30 healthy controls. The clustering of lncRNAs placed in entire samples. B, Pairwise comparison of lncRNAs in the training set. Normal samples were considered as controls, and their expression level values were considered as 1. The Mann‐Whitney *U* test was used to assess the differences of LncRNAs level between groups. The relative expression was considered as significant when <0.5 or >2

It has been proven that tumor lncRNAs boost or suppress the CRC progress, but their functions in other tumor environment cells have not been elucidated properly.^4^ We have chosen 30 known cancer‐related lncRNAs and evaluated their levels in 60 fecal samples obtained from cases with normal and cancer colons. The normal group was chosen as control. Considering the relative expression <0.5 or >2, we found 10 differentially expressed lncRNAs (DElncRNAs; Table 3; Figure 1). The combination of these 10 DElnRNAs was selected as the predictive panel for further analysis.

**Table 3:** The list of differentially expressed fecal lncRNAs (DElncRNAs) in training set

Differentially expressed lncRNAs	Number of DElncRNAs	Name of DElncRNAs (fold change)
Up‐regulated lncRNAs	7	CCAT1 (4.5) CCAT2 (2.8) H19 (2.1) HOTAIR (3.2) HULC (2.4) MALAT1 (2.7) PCAT1 (2.5)
Down‐regulated lncRNAs	3	MEG3 (0.4) PTENP1 (0.3) TUSC7 (0.5)

Note

The fold change criteria were set as >2 or <0.5.

### 3.3 Establishment and validation of the predictive panel

**Figure 2:**
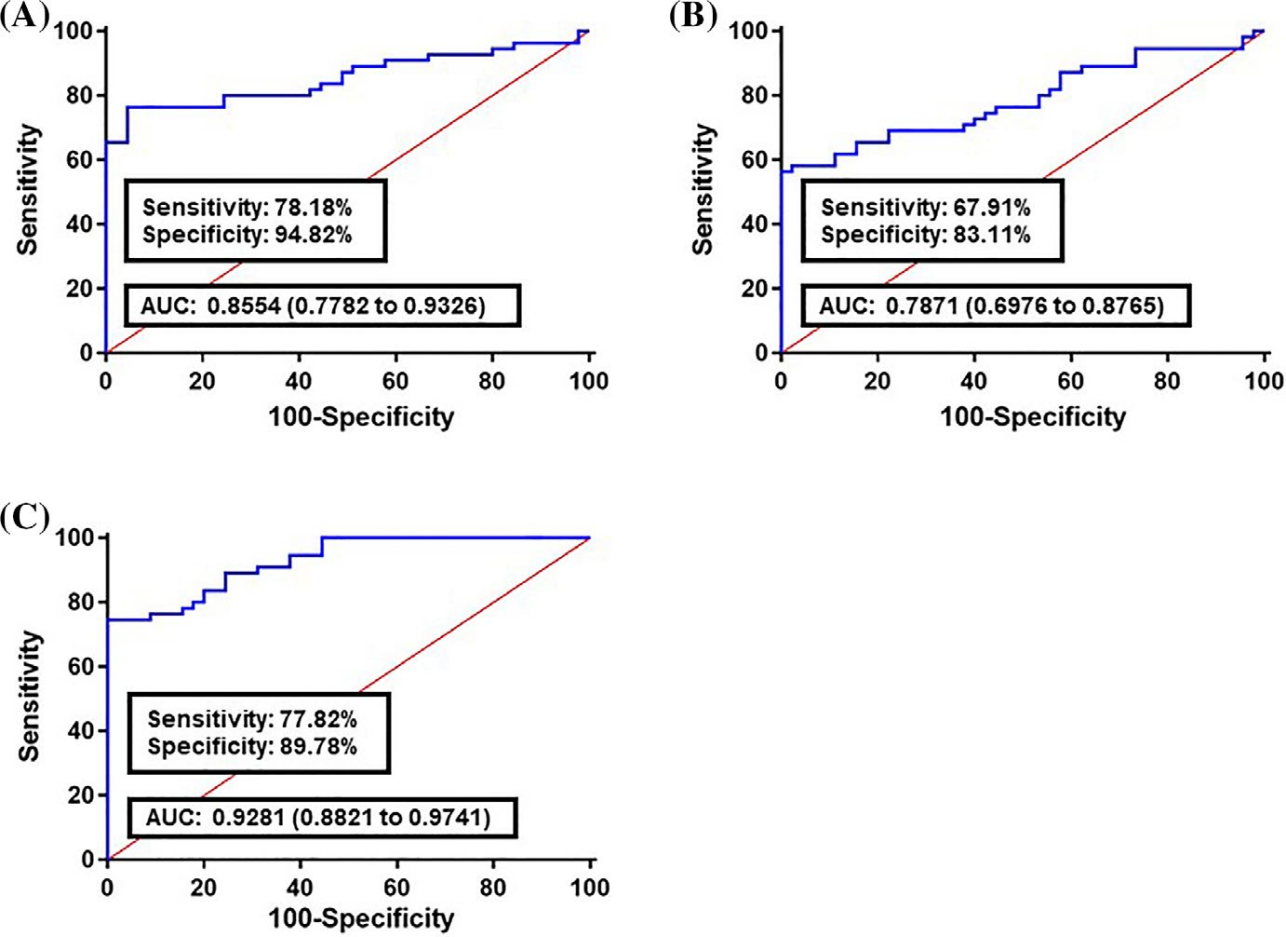
Receiver operating characteristics (ROC) curve analysis of the logit model with fecal lncRNAs CCAT1, CCAT2, H19, HOTAIR, HULC, MALAT1, MEG3, PCAT1, PTENP1, and TUSC7, on the training set. A, The area under the ROC curve (AUC) assessment of the logit(*p*) value for the fecal lncRNAs panel in istinguishing colorectal cancer (CRC) cases (All TNM stages) from the healthy controls. B, AUC assessment of the logit(*p*) value for the fecal lncRNAs panel in distinguishing the early CRC stages (I‐II TNM stages) from the healthy controls. C, AUC assessment of the logit(*p*) value for the fecal lncRNAs panel in distinguishing the advanced CRC stages (III‐IV TNM stages) from the healthy controls. logit(*p*) = 27.886 − 0.1969 × (CCAT1) − 0.1904 × (CCAT2) − 0.2986 × (H19) − 0.6895 × (HOTAIR) − 0.1194 × (HULC) − 0.3598 × (MALAT1) − 0.1278 × (MEG3) − 0.1744 × (PCAT1) − 0.9081 × (PTENP1) − 0.8469 × (TUSC7)

**Figure 3:**
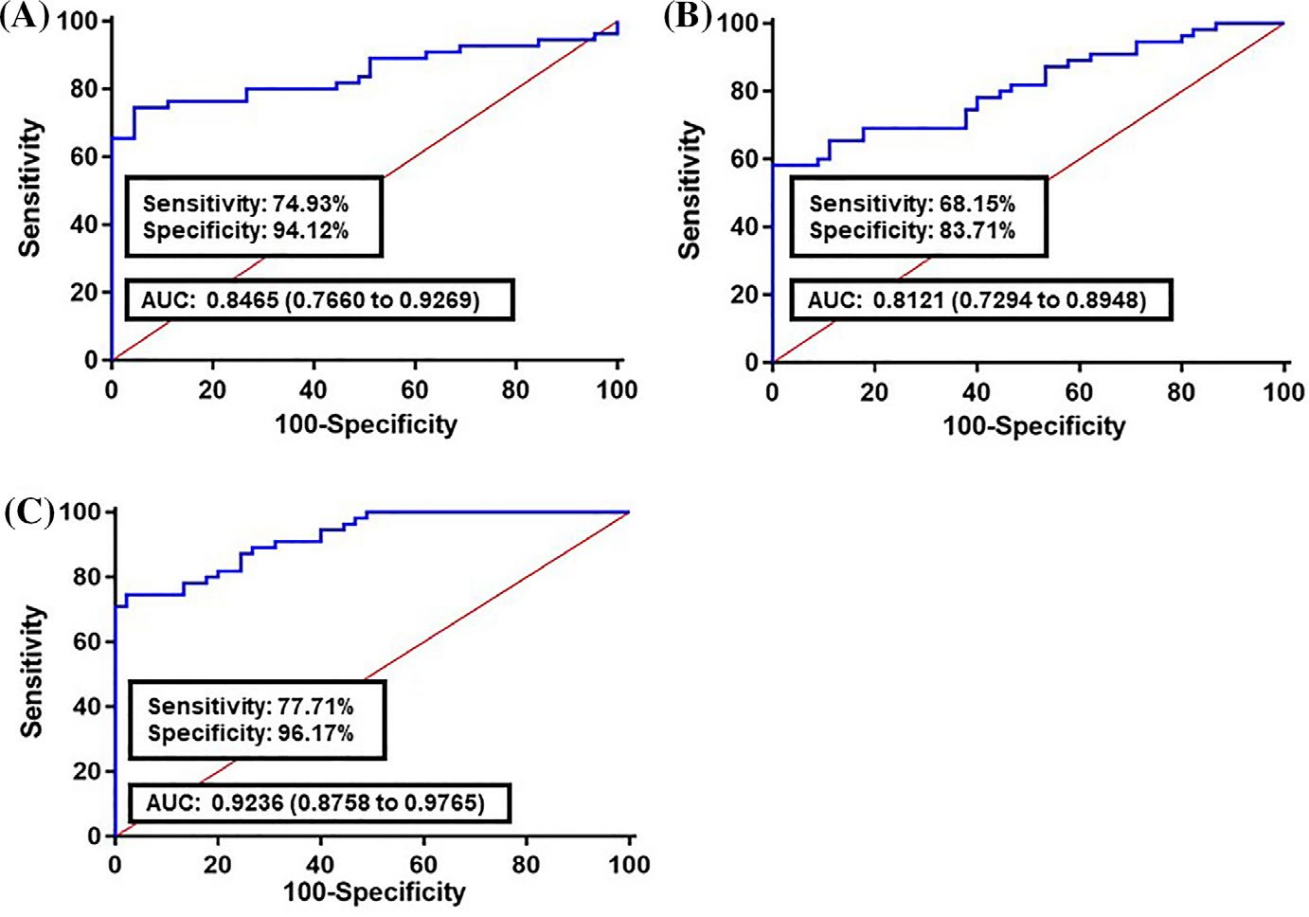
Receiver operating characteristics (ROC) curve analysis of the logit model with fecal lncRNAs CCAT1, CCAT2, H19, HOTAIR, HULC, MALAT1, MEG3, PCAT1, PTENP1, and TUSC7, on the validation set. A, The area under the ROC curve (AUC) assessment of the logit(*p*) value for the fecal lncRNAs panel in distinguishing colorectal cancer (CRC) cases (All TNM stages) from the healthy controls. B, AUC assessment of the logit(*p*) value for the fecal lncRNAs panel in distinguishing the early CRC stages (I‐II TNM stages) from the healthy controls. C, AUC assessment of the logit(*p*) value for the fecal lncRNAs panel in distinguishing the advanced CRC stages (III‐IV TNM stages) from the healthy controls. logit(*p*) = 27.886 − 0.1969 × (CCAT1) − 0.1904 × (CCAT2) − 0.2986 × (H19) − 0.6895 × (HOTAIR) − 0.1194 × (HULC) − 0.3598 × (MALAT1) − 0.1278 × (MEG3) − 0.1744 × (PCAT1) − 0.9081 × (PTENP1) − 0.8469 × (TUSC7)

A logistic regression model was built based on the comparison of normal and cancer colon fecal samples to estimate the risk of a patient being diagnosed with CRC. Our analysis demonstrated that all 10 long non‐coding RNAs were significant predictors (Table 4). The ROC curve was built using the logit model of candidate long non‐coding RNAs as follows: logit (*p*) = 27.886 − 0.1969 × (CCAT1) − 0.1904 × (CCAT2) − 0.2986 × (H19) − 0.6895 × (HOTAIR) − 0.1194 × (HULC) − 0.3598 × (MALAT1) − 0.1278 × (MEG3) − 0.1744 × (PCAT1) − 0.9081 × (PTENP1) − 0.8469 × (TUSC7). Compared with the normal group, the AUC value for the long non‐coding RNAs panel was 0.8554 in all CRC stages (I‐IV TNM stages) along with 78.18% sensitivity and 94.82% specificity (Figure 2A). For early CRC stages (I‐II TNM stages), the AUC was 0.7871 with 67.91% sensitivity and 83.11% specificity (Figure 2B). Analyzing the advanced CRC stages (III‐IV TNM stages) showed that the AUC was 0.9281 with 77.82% sensitivity and 89.78% specificity (Figure 2C). To validate the diagnostic performance of the panel, we examined it in an independent set, including 30 CRC and 30 normal samples. In comparison with healthy subjects, the AUC value for the lncRNA panel was 0.8465 in all CRC stages (I‐IV TNM stages) along with 74.93% sensitivity and 94.24% specificity (Figure 3A). For early CRC stages (I‐II TNM stages), the AUC was 0.8121 with 68.15% sensitivity and 83.71% specificity (Figure 3B). Analyzing advanced CRC stages (III‐IV TNM stages) showed that the AUC was 0.9236 with 77.71% sensitivity and 96.17% specificity (Figure 3C).

**Table 4:** Expression analysis and diagnostic performance of DElncRNAs in training cohort

lncRNA	Sensitivity	Specificity	Ct	Youden's index *J*	AUC	*P* value	95% CI
CCAT1	92.73	93.33	≤25	0.8606	0.7034	6.3 E−04	0.5212~0.8056
CCAT2	80	80	≤22	0.6	0.6416	3.3 E−06	0.5335~0.7497
H19	90.91	88.89	≤26	0.798	0.5584	4.1 E−06	0.4452~0.6715
HOTAIR	94.55	93.17	≤25	0.8772	0.6408	4.7 E−06	0.5333~0.7483
HULC	92.73	91.11	≤27	0.8384	0.6372	6.1 E−04	0.5283~0.7461
MALAT1	81.82	77.78	≤22	0.596	0.6331	4.4 E−05	0.525~0.7413
MEG3	89.09	88.89	≤26	0.7798	0.6638	5.9 E−03	0.5575~0.7701
PCAT1	92.73	93.33	≤25	0.8606	0.5584	1.2 E−05	0.4445~0.6723
PTENP1	90.91	92.15	≤27	0.8306	0.5875	3.1 E−03	0.4742~0.7007
TUSC7	81.82	82.22	≤26	0.6404	0.6339	2.5 E−04	0.5253~0.7426

Note

Our results showed that all the 10 long non‐coding RNAs were significant predictors.

### 3.4 Analyzing the predictive panel between CRC and polyp cases

**Figure 4:**
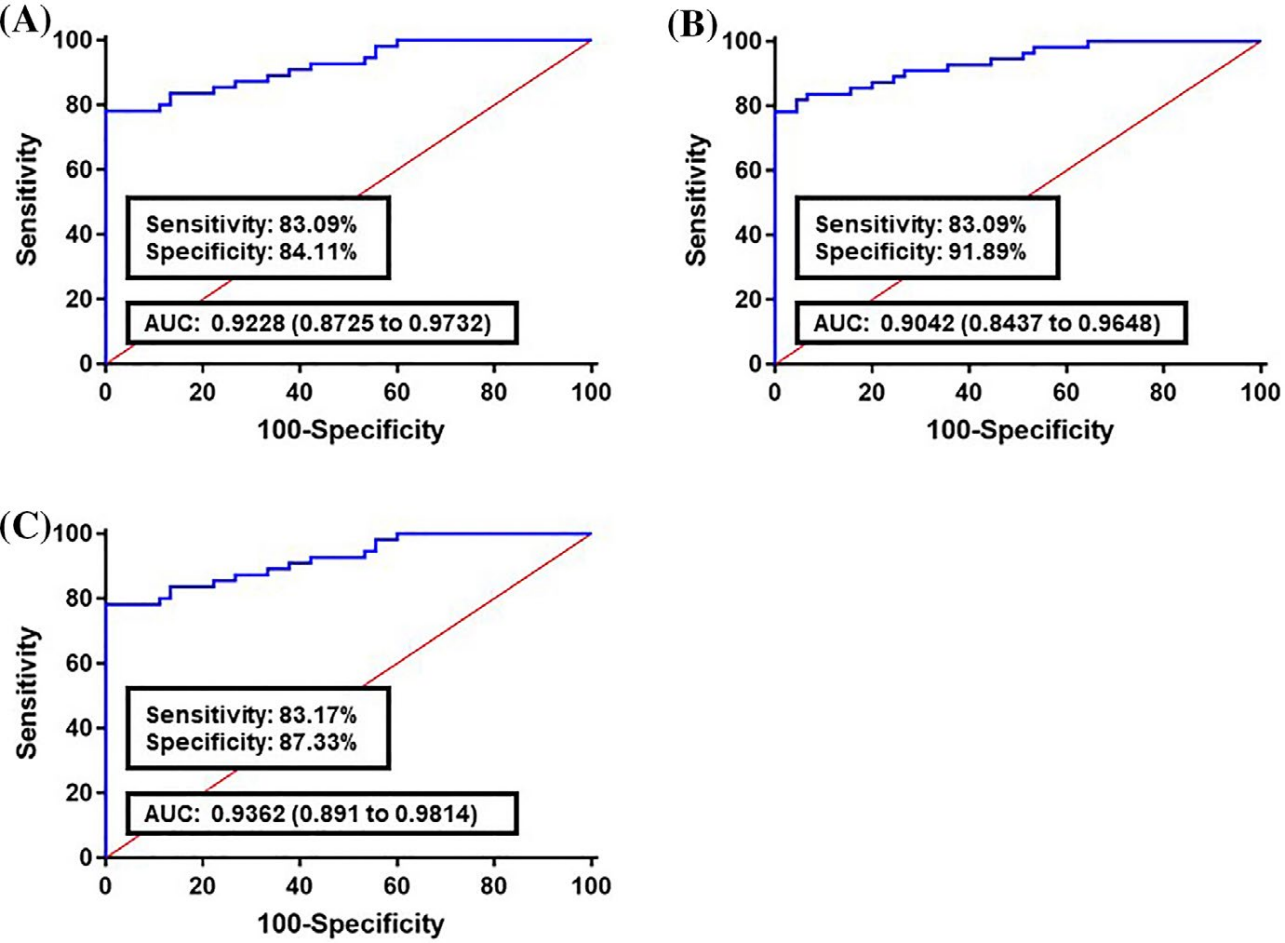
Receiver operating characteristics (ROC) curve analysis of the logit model with fecal lncRNAs CCAT1, CCAT2, H19, HOTAIR, HULC, MALAT1, MEG3, PCAT1, PTENP1, and TUSC7, in the comparison of colorectal cancer (CRC) cases with individuals with colon polyps. A, The area under the ROC curve (AUC) assessment of the logit(*p*) value for the fecal lncRNAs panel in distinguishing the CRC cases (All TNM stages) from the polyp group. B, AUC assessment of the logit(*p*) value for the fecal lncRNAs panel in distinguishing the early CRC stages (I‐II TNM stages) from the polyp group. C, AUC assessment of the logit(*p*) value for the fecal lncRNAs panel in distinguishing the advanced CRC stages (III‐IV TNM stages) from the polyp group. logit(*p*) = 27.886 − 0.1969 × (CCAT1) − 0.1904 × (CCAT2) − 0.2986 × (H19) − 0.6895 × (HOTAIR) − 0.1194 × (HULC) − 0.3598 × (MALAT1) − 0.1278 × (MEG3) − 0.1744 × (PCAT1) − 0.9081 × (PTENP1) − 0.8469 × (TUSC7)

The diagnostic power of the ten‐DElncRNA panel was further estimated between CRC samples from the training cohorts and stools obtained from people with colon polyps (Figure 4). The corresponding AUC for CRC stages (I‐IV TNM stages), (I‐II TNM stages), and (III‐IV TNM stages) were 0.9228, 0.9042, and 0.9362, respectively. These results show that, in comparison with normal vs CRC samples, our panel has a higher sensitivity and specificity for polyp transition into CRC status.

### 3.5 Functional annotations of validated lncRNAs

We performed a gene set enrichment analysis (GSEA) of validated lncRNAs based on target molecules and the type of interactions. Our final panel consisted of seven up‐regulated DElncRNAs (CCAT1, CCAT2, H19, HOTAIR, HULC, MALAT1, PCAT1), which were also known as oncolncRNAs and three down‐regulated lncRNAs (MEG3, PTENP1, and TUSC7) as tumor suppressor lncRNAs (tslncRNAs). Using NPInter algorithms, the DNA, RNA (mRNA and miRNA), and protein targets of intended lncRNAs were extracted from previously published literature reports. Using the Cytoscape plugin ClueGO, the nodes were networked according to their interaction type. The Cytoscape plugin Cyto‐Hubba was applied to identify the targets with the highest degree of interactions shared between multiple lncRNAs and miRNAs and proteins. To identify the biological functions, the groups were submitted to the EnrichR tool. The enrichment analysis results of intended prognostic lncRNAs showed that they might participate in CRC tumorigenesis through four different interactions.

## 4 DISCUSSION

Identifying of the lncRNAs that are effective in the development of cancer requires the examination of samples in the early phases of the formation of malignancy (such as colon polyps) and their comparison with the healthy and patient groups. The researchers tended to examine the types of biological samples of those that were low‐cost, non‐invasive, and accessible to all individuals, such as blood plasma.^9^ The problem with the use of blood plasma is its circulation through all tissues of the body and secretion of various cellular products into the blood. This makes it difficult to detect actual cancer biomarkers. The stool only passes through the intestines and rectum and is much less polluted compared to the blood plasma; therefore, it is suitable for examining the status of CRC markers in different groups, including patients, individuals suspected of being malignant and healthy people.

There has been no previous study on lncRNAs expression alteration between CRC patients and healthy individuals, so we have examined a panel of 30 with cancer‐related lncRNAs in stool specimens. Our results showed that the lncRNA expression index was highly capable of distinguishing the cancer patients from the controls with a lesser magnitude. The results of the expression analysis of the 10 fecal DElncRNAs are in agreement with the previous literature reports of these lncRNAs in CRC cases. For example, OncolncRNAs CCAT1 and CCAT2 are found greater in all stages of colon cancer and associated with tumor stage, recurrence‐free survival, and overall survival of CRC patients.^14^

The lncRNA H19 gene is located on human chromosome 11p15.5 and is involved in the carcinogenesis, progression, and metastasis of CRC.^15^ Up‐regulation of oncolncRNA H19 correlates with tumor differentiation, the TNM stage, and poor prognosis of colon cancer.^16^ OncolncRNA HOTAIR overexpression is associated with tumor invasion, metastasis, tumor differentiation, tumor stage, and vascular invasion.^17^ The 13 cancer‐related lncRNAs panel showed that combined evaluation of plasma CCAT1 and HOTAIR had a good diagnostic performance for CRC screening, especially in early CRC, and provides a more effective diagnosis performance than HOTAIR or CCAT1 alone in plasma and serum samples of CRC patients (AUC = 0.954, *P* < .001, sensitivity, 84.3%; specificity, 80.2%).^18^ Using the Cytoscape plugin Cyto‐Hubba, we identified the targets with the highest degree of interactions shared between multiple lncRNAs. As in RNA‐protein category, six proteins including FUS/TLS, HNRNPA2B1, p53, PRC2, Upf1, and WDR33 were shared between H19, HOTAIR, HULC, MEG3, MALAT1, PTENP1, and PCAT1 lncRNAs. Notably, these proteins possess multiple tasks in cells and thereby may associate with various factors. For example, FUS/TLS is an hnRNP family member with an RNA recognition motif (RRM) for RNA interaction and has three arginine‐glycine‐glycine‐rich (RGG) motifs for binding to proteins. Using this structure, FUS/TLS is able to participate in various biological functions, including DNA repair, transcription, pre‐mRNA splicing, miRNA processing, interacting with lncRNAs, mRNA stability, mRNA transport, and mRNA translation.^19^ Overexpression of FUS/TLS was reported in sporadic CRC cells^20^ and associates with tumorigenesis and metastasis in lung cancer through E‐cadherin down‐regulation.^21^ The direct associations, until today, between lncRNAs MALAT1 and NEAT1‐2 with FUS/TLS and TDP‐43 have been proved in ALS/FTLD patients.^22^ The other hnRNP family member, HNRNPA2B1, acts as a mediator of m(6)A‐dependent nuclear RNA and contributes to pre‐mRNA splicing in the nucleus. The aberrant level of HNRNPA2B1 has been shown in colon and gastric cancers.^23^ Most of the received NPInter RNA type targets were miRNAs (data not shown). From this list, 39 miRNAs were shared between H19, HOTAIR, MALAT, MEG3, and PCAT1. Referring to the published series of CRC‐related GEO datasets including GSE35834,^24^ GSE54088,^25^ and GSE39845,^26^ we found that 34 miRNAs of our list have been previously reported as DEmiRNAs in colon cancer. Among them, only the fecal level of hsa‐miR‐17‐5p and hsa‐miR‐29a‐3p was previously investigated in CRC patients and both were miRNAs associated with tumor location.^27^, ^28^ As there are no other investigations on the underlined regulatory networks of these miRNAs in CRC initiation and progression, our findings could be considered as a step forward in better understanding cancer regulatory structures. An overall genome mapping study of cancer associated lncRNAs MALAT1 and NEAT1 in MCF‐7 breast cancer cells identified these genes as possible targets of MALAT1.^29^ It has been shown also that knocking down MALAT1 in CaSki cervical cancer cells increased proliferation and invasion rates through BAX up‐regulation.^30^ MALAT1 could interact and up‐regulate the pre‐mRNA factors SRSF1 and PRPF6, and PRPF6 acts as a splicing regulatory of MALAT1.^31^ Similar interactions have been reported between ZFP36 and MALAT1, whereas MALAT1 sequence has a regulatory binding site for ZFP36,^32^ and MALAT1 overexpresses ZFP36.^29^ The oncolncRNA HOTAIR gene is located within the HOXD gene clusters and has a negative effect on other HOXD clusters, being placed on the other chromosomes.^33^ Evidence suggests that HOTAIR may target the HOXD cluster genes at RNA and protein levels.^34^ It could also repress the HOXD genes in an alternative manner by targeting the polycomb repressive complex 2 (PRC2) family member SUZ12 and induces gene silencing through H3K27 methylation and H3K4 demethylation.^35^

## 5 CONCLUSION

Our study, for the first time, examined the possibility of lncRNAs evaluation in human stools and introduced a panel based on cancer‐related lncRNAs that could identify and distinguish CRC patients from healthy individuals or those with polyps. In addition, our results showed that the measurement of cancer‐related lncRNAs as a panel had more sensitivity and specificity than those lncRNAs alone. Finally, a comprehensive range of lncRNAs should be measured to further elaborate on their regulatory network.

## CONFLICT OF INTEREST

The authors have declared that no competing interests exist.

## AUTHOR CONTRIBUTION

Ehsan Nazemalhosseini‐Mojarad involved in study conception and design. Ehsan Gharib, Kaveh Baghdar, Zahra Nayeri, Hossein Sadeghi, Sama Rezasoltani, Arezo Jamshidi‐Fard, Pegah Larki, Amir Sadeghi, Mehrdad Hashemi, and Hamid Asadzadeh Aghdaei involved in acquisition of data. Ehsan Nazemalhosseini‐Mojarad analyzed and interpreted the data. Ehsan Gharib and Ehsan Nazemalhosseini‐Mojarad drafted the study.
